# Supplementary material for: The mutREAD method detects mutational signatures from low quantities of cancer DNA
Source: Nat Commun. 2020 Jun 23;11:3166. doi: 10.1038/s41467-020-16974-3 (PMC7311535; doi:10.1038/s41467-020-16974-3)
Supplement: Supplementary file 1 — Supplementary Information [file 41467_2020_16974_MOESM1_ESM.pdf]

Supplementary Information for:

# The mutREAD method detects mutational signatures from low quantities of cancer DNA

Perner et al.

# Supplementary Figures

A

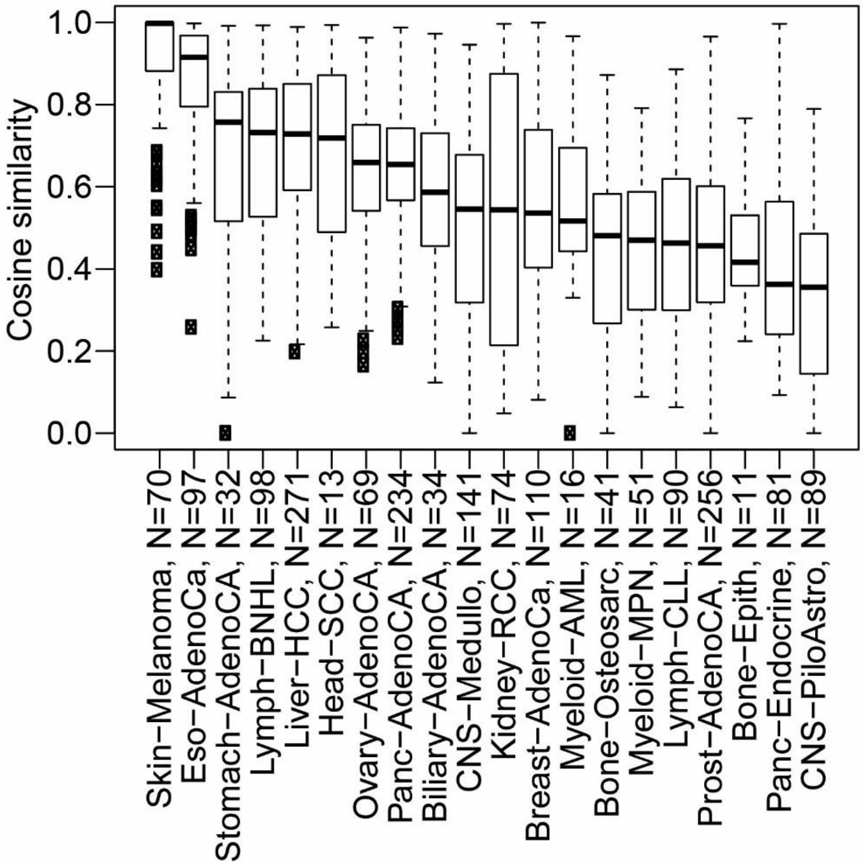

Cosine similarity
1.0
0.8
0.6
0.4
0.2
0.0
Skin−Melanoma, N=70
Eso−AdenoCa, N=97
Stomach−AdenoCA, N=32
Lymph−BNHL, N=98
Liver−HCC, N=271
Head−SCC, N=13
Ovary−AdenoCA, N=69
Panc−AdenoCA, N=234
Biliary−AdenoCA, N=34
CNS−Medullo, N=141
Kidney−RCC, N=74
Breast−AdenoCa, N=110
Myeloid−AML, N=16
Bone−Osteosarc, N=41
Myeloid−MPN, N=51
Lymph−CLL, N=90
Prost−AdenoCA, N=256
Bone−Epith, N=11
Panc−Endocrine, N=81
CNS−PiloAstro, N=89


B

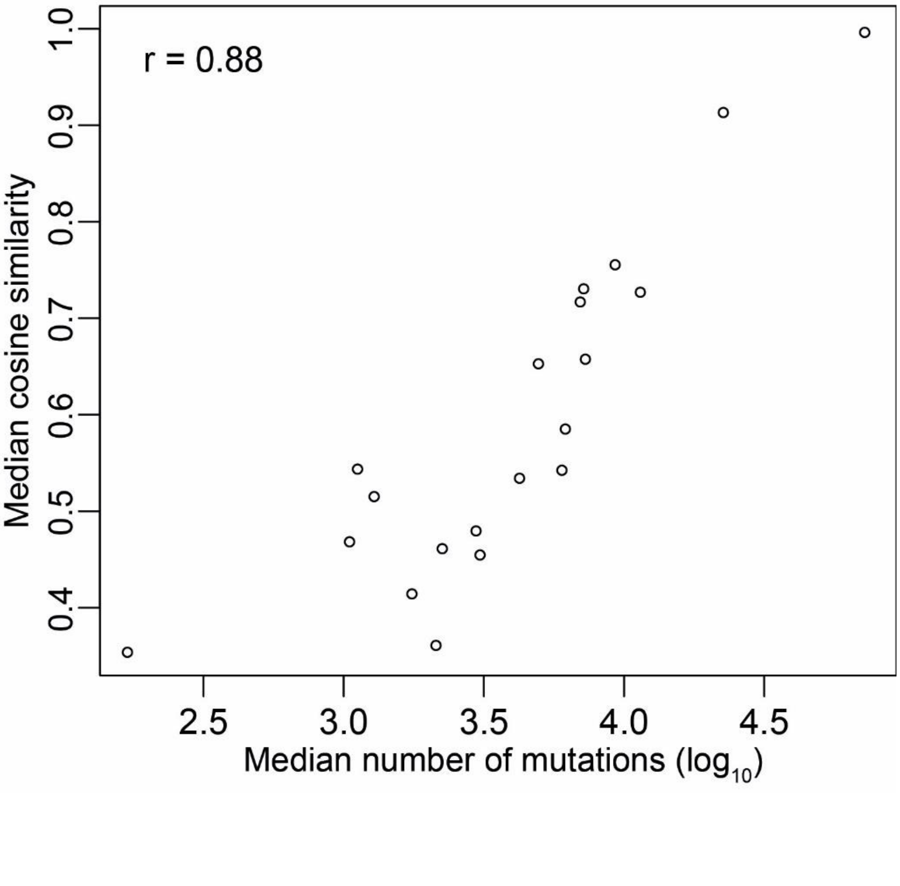

r = 0.88
Median cosine similarity
1.0
0.9
0.8
0.7
0.6
0.5
0.4
2.5
3.0
3.5
4.0
4.5
Median number of mutations ($log_{10}$)

C

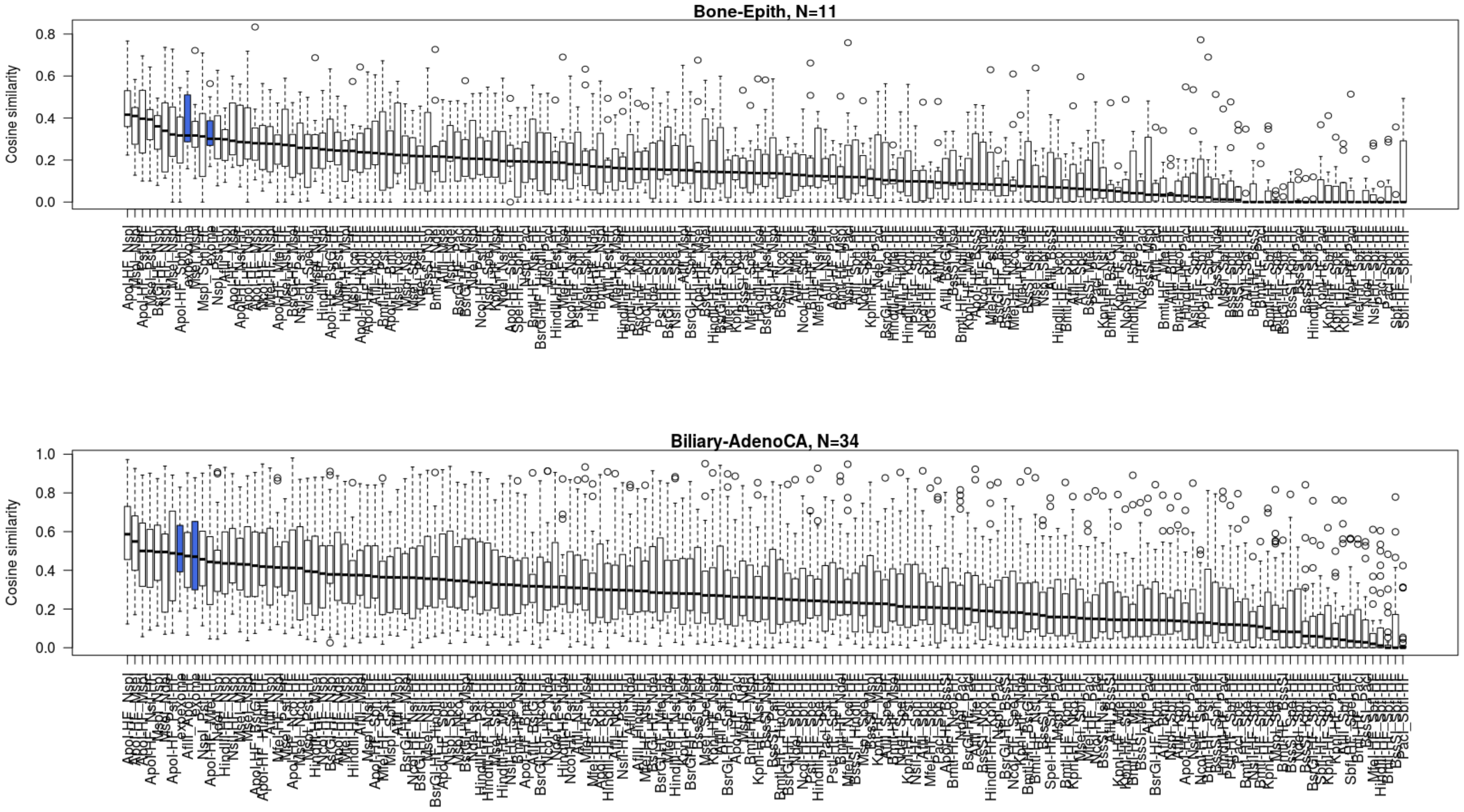

Bone-Epith, N=11
Cosine similarity
Biliary-AdenoCA, N=34
Cosine similarity

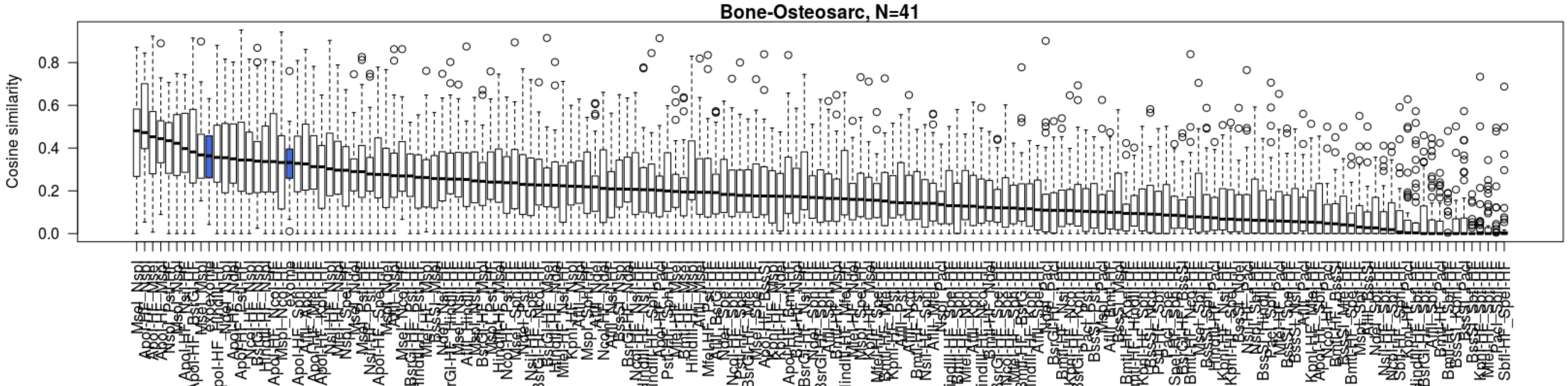
Bone-Osteosarc, N=41
Cosine similarity
0.0
0.2
0.4
0.6
0.8

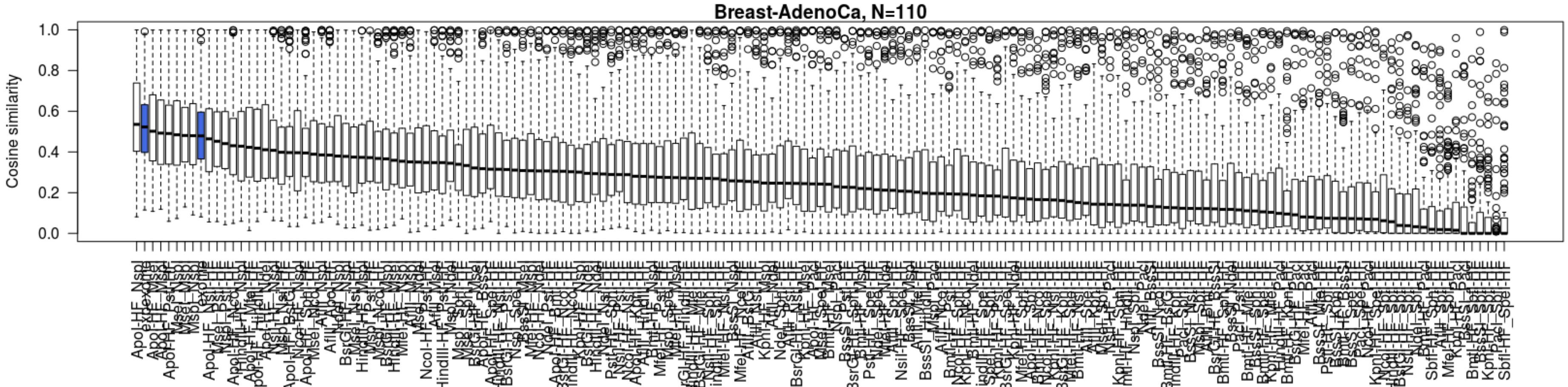
Breast-AdenoCa, N=110
Cosine similarity
0.0
0.2
0.4
0.6
0.8
1.0

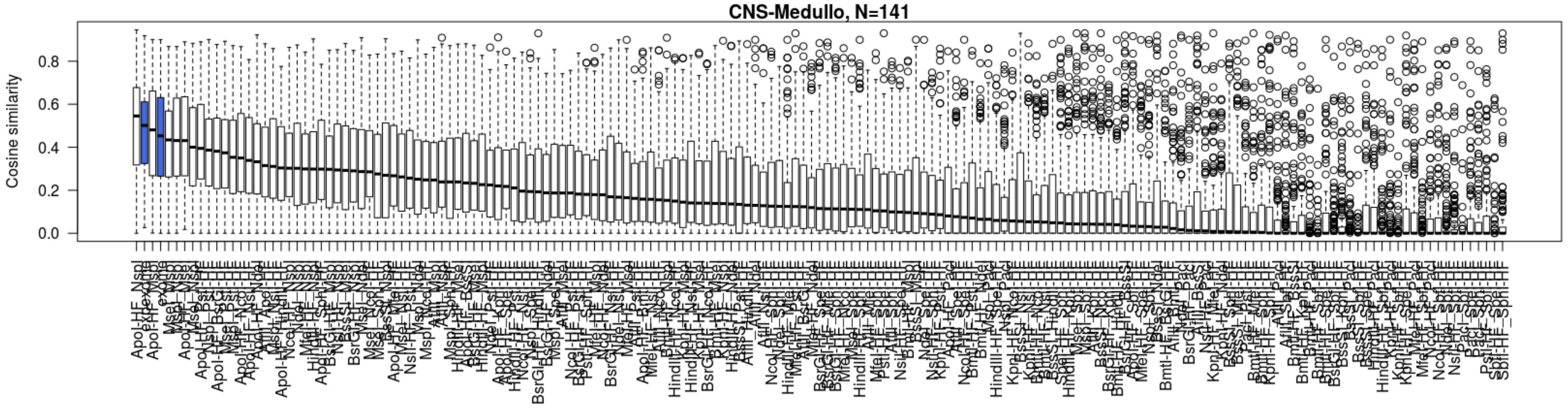
CNS-Medullo, N=141
Cosine similarity
0.0
0.2
0.4
0.6
0.8

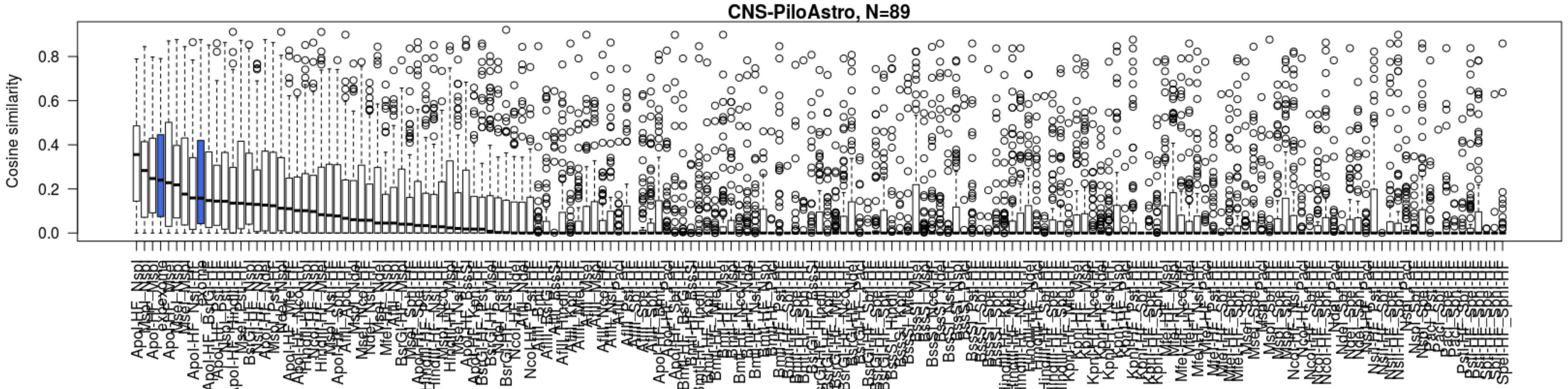
CNS-PiloAstro, N=89
Cosine similarity
0.0
0.2
0.4
0.6
0.8

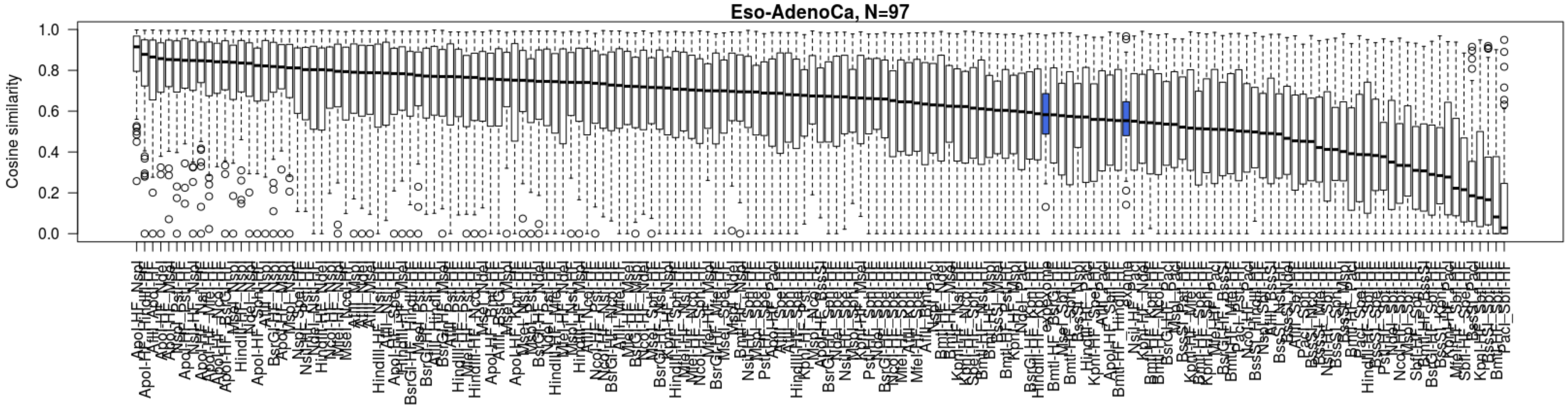
Eso-AdenoCa, N=97
Cosine similarity
1.0
0.8
0.6
0.4
0.2
0.0

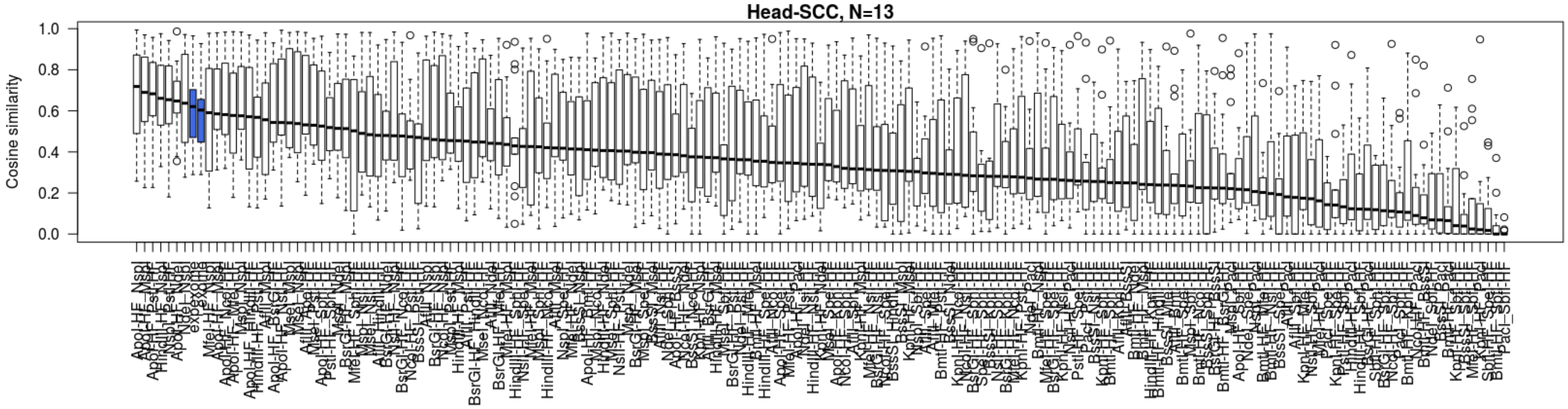
Head-SCC, N=13
Cosine similarity
1.0
0.8
0.6
0.4
0.2
0.0

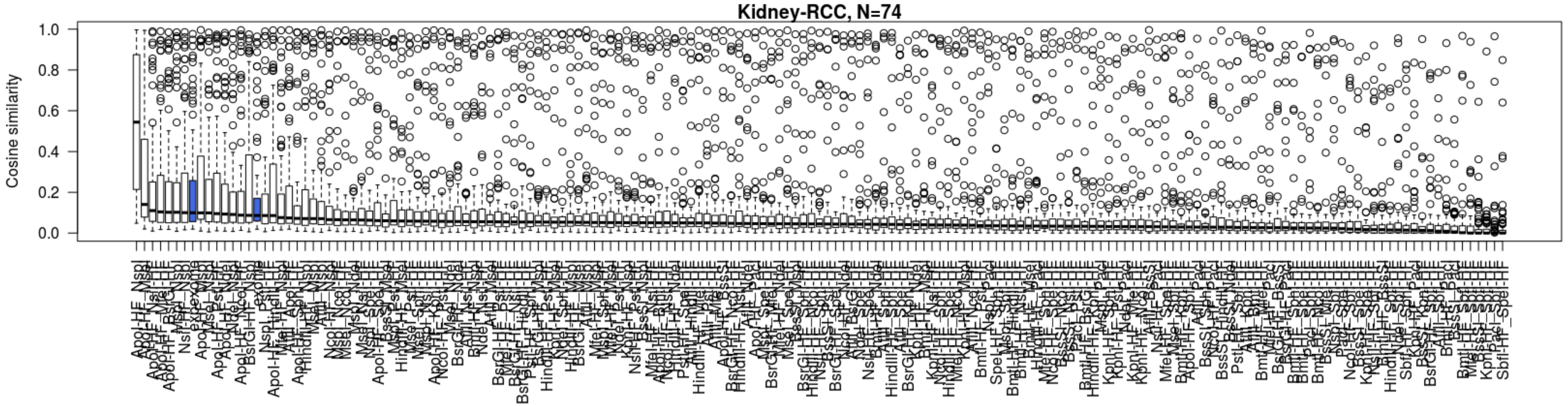
Kidney-RCC, N=74
Cosine similarity
0.0
0.2
0.4
0.6
0.8
1.0

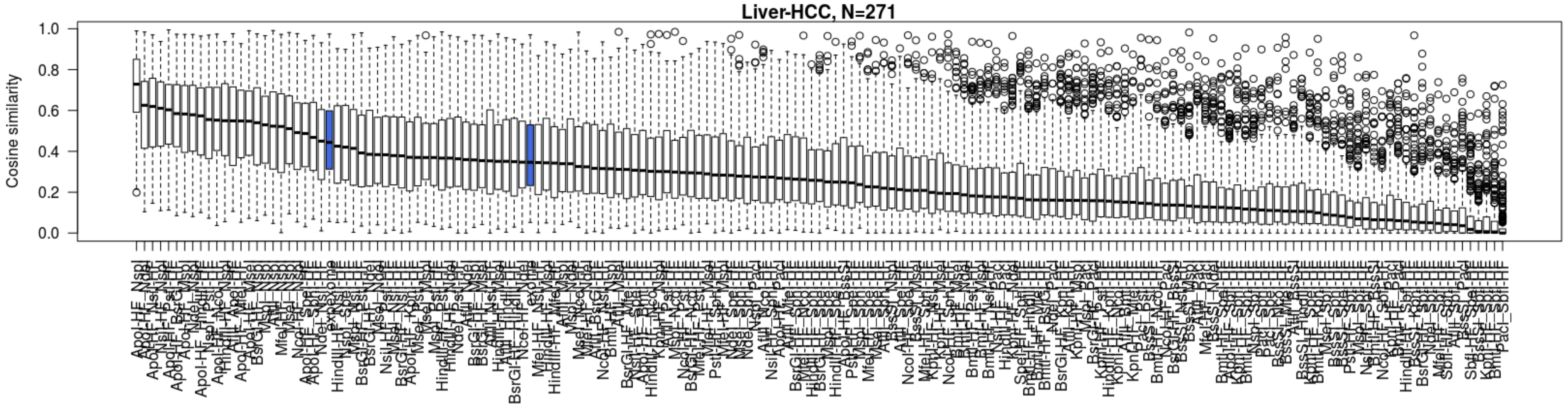
Liver-HCC, N=271
Cosine similarity
0.0
0.2
0.4
0.6
0.8
1.0

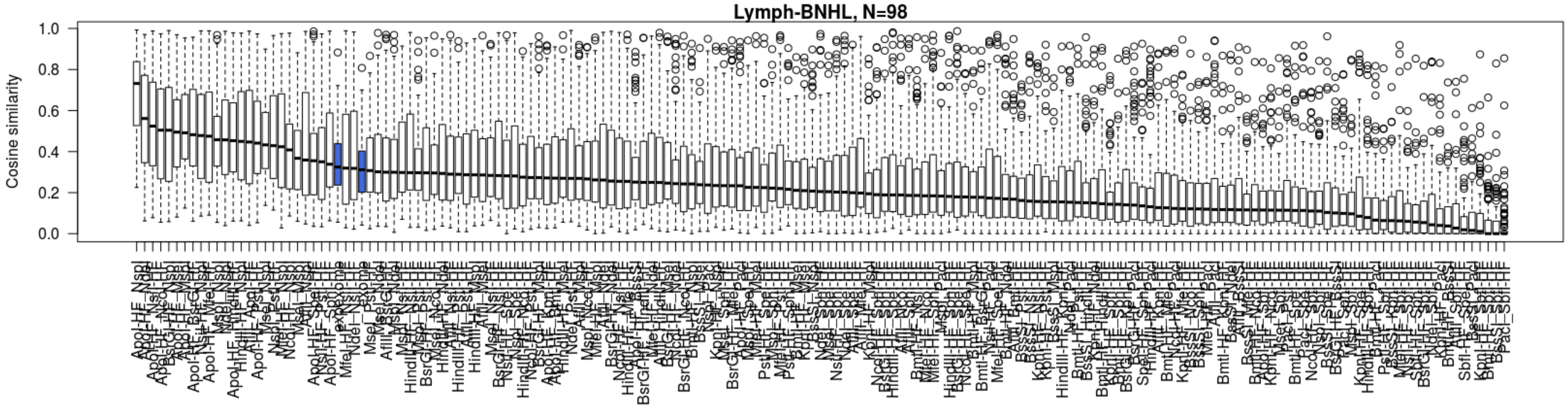
Lymph-BNHL, N=98
Cosine similarity
0.0
0.2
0.4
0.6
0.8
1.0

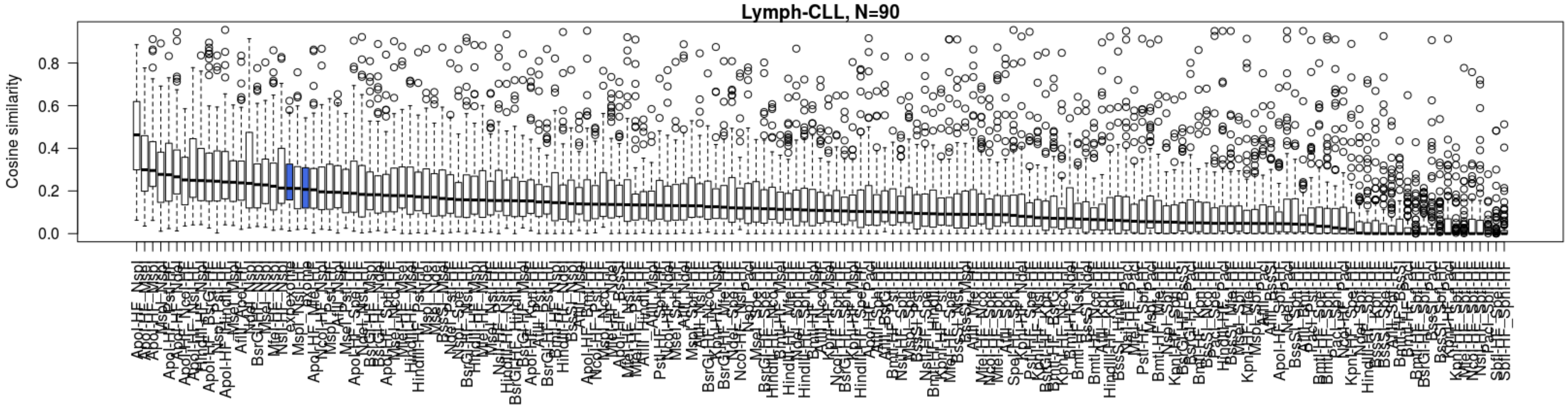
Lymph-CLL, N=90
Cosine similarity
0.0
0.2
0.4
0.6
0.8

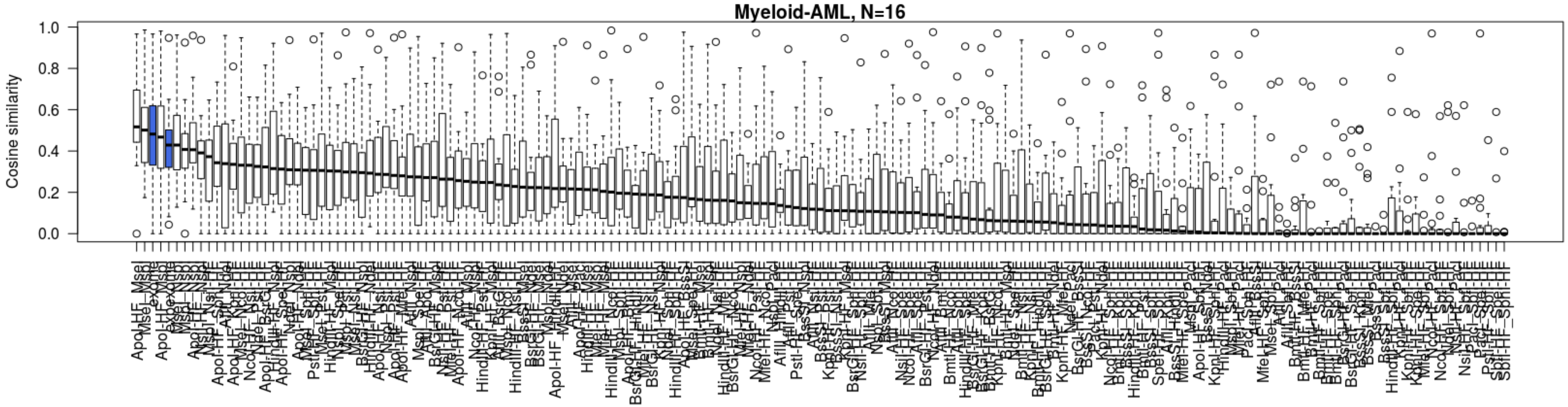
Myeloid-AML, N=16
Cosine similarity
1.0
0.8
0.6
0.4
0.2
0.0

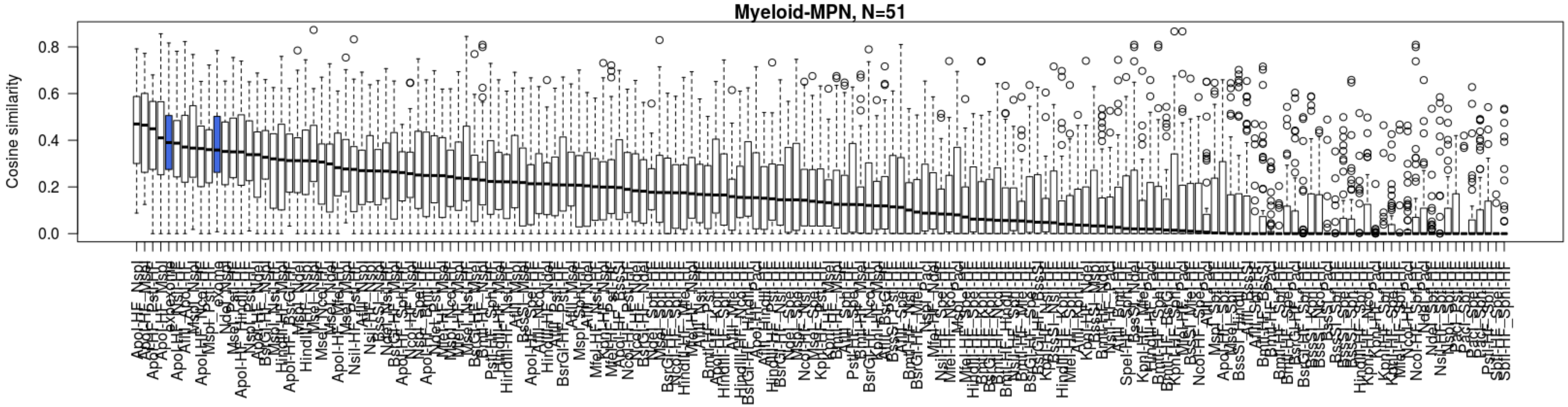
Myeloid-MPN, N=51
Cosine similarity
0.8
0.6
0.4
0.2
0.0

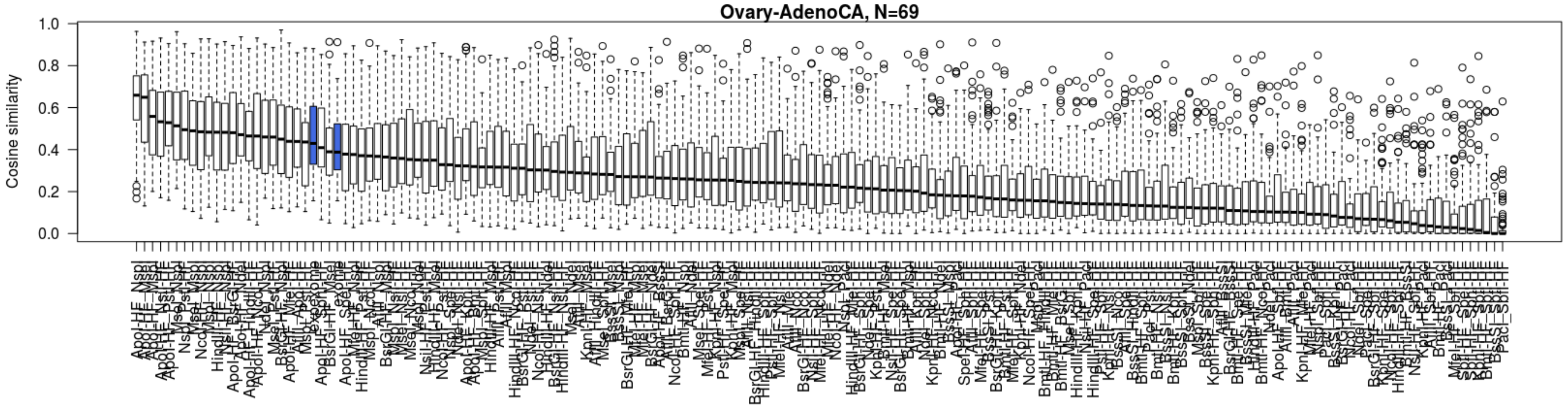
Ovary-AdenoCA, N=69
Cosine similarity
1.0
0.8
0.6
0.4
0.2
0.0

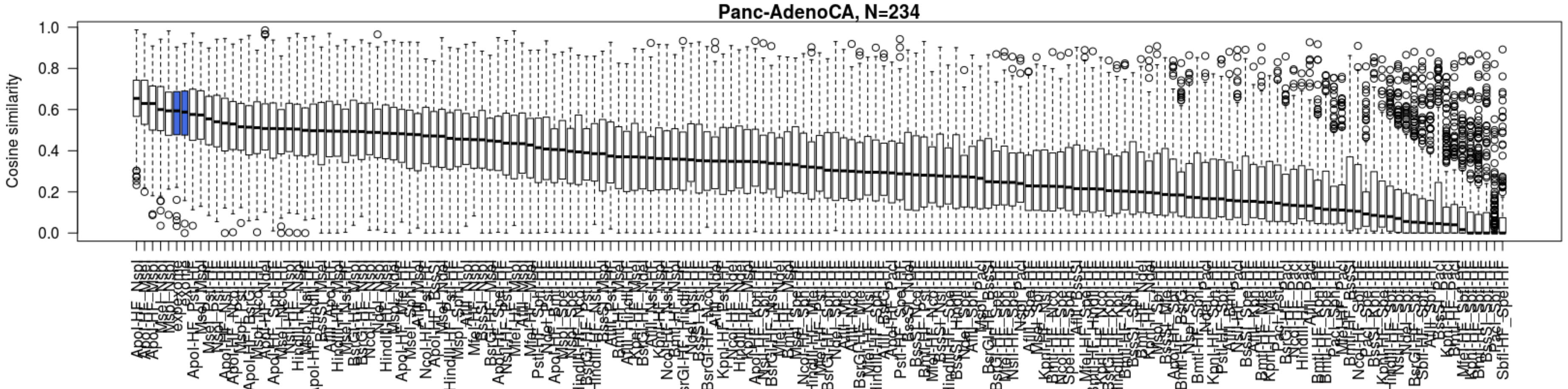
Panc-AdenoCA, N=234
Cosine similarity
1.0
0.8
0.6
0.4
0.2
0.0

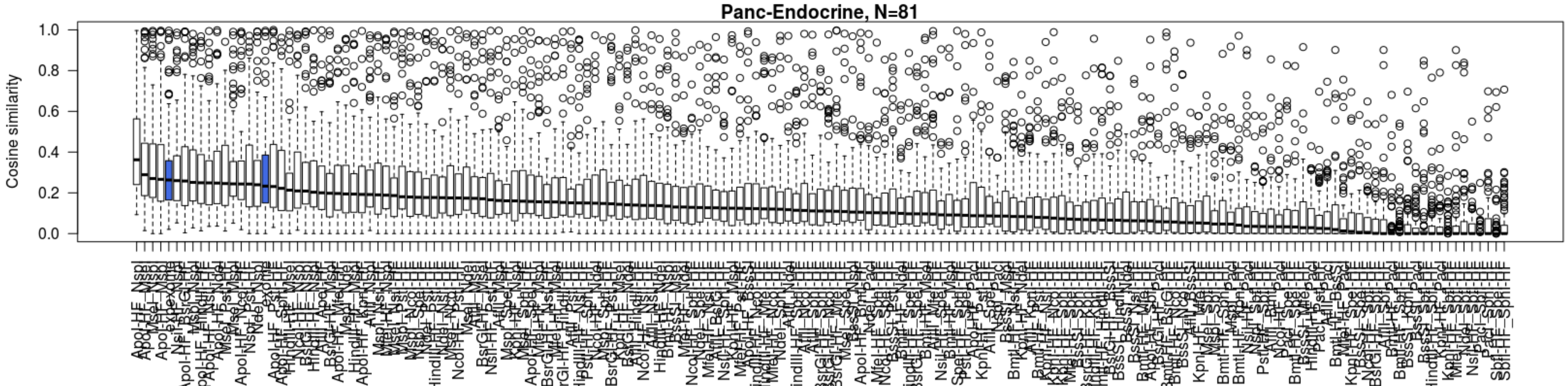
Panc-Endocrine, N=81
Cosine similarity

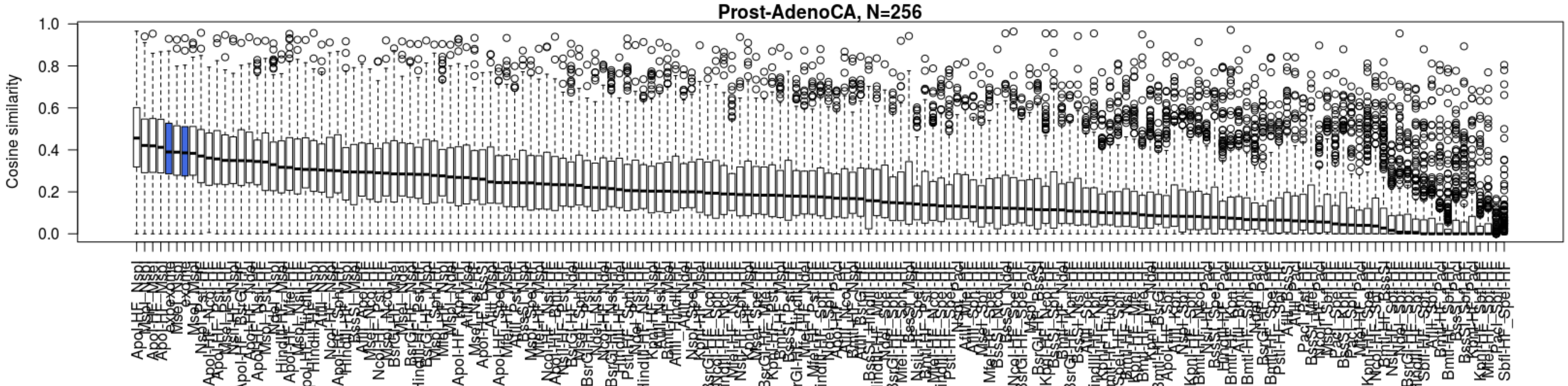
Prost-AdenoCA, N=256
Cosine similarity

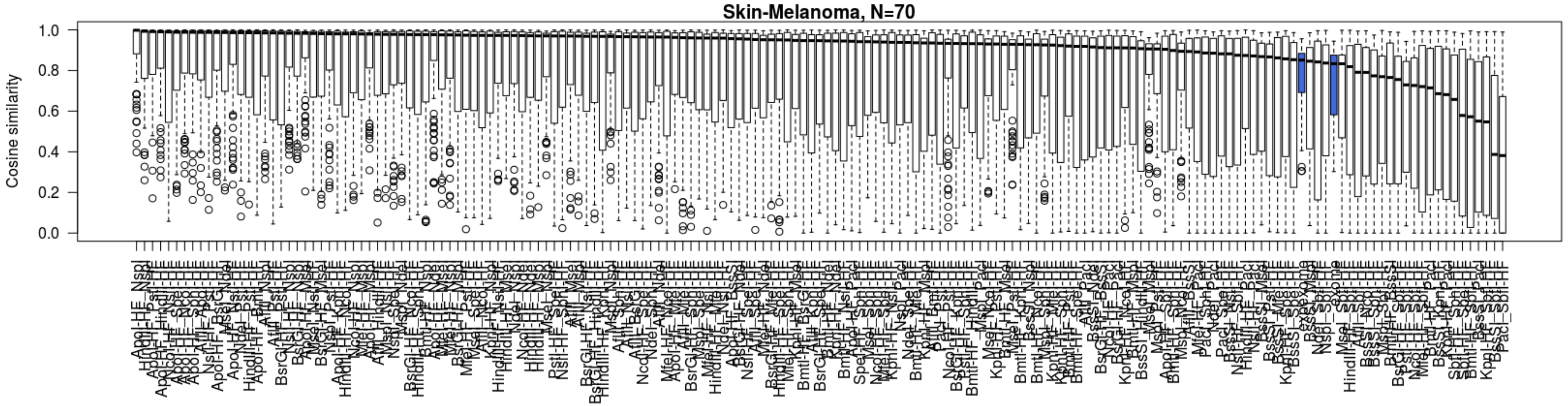
Skin-Melanoma, N=70
Cosine similarity
1.0
0.8
0.6
0.4
0.2
0.0

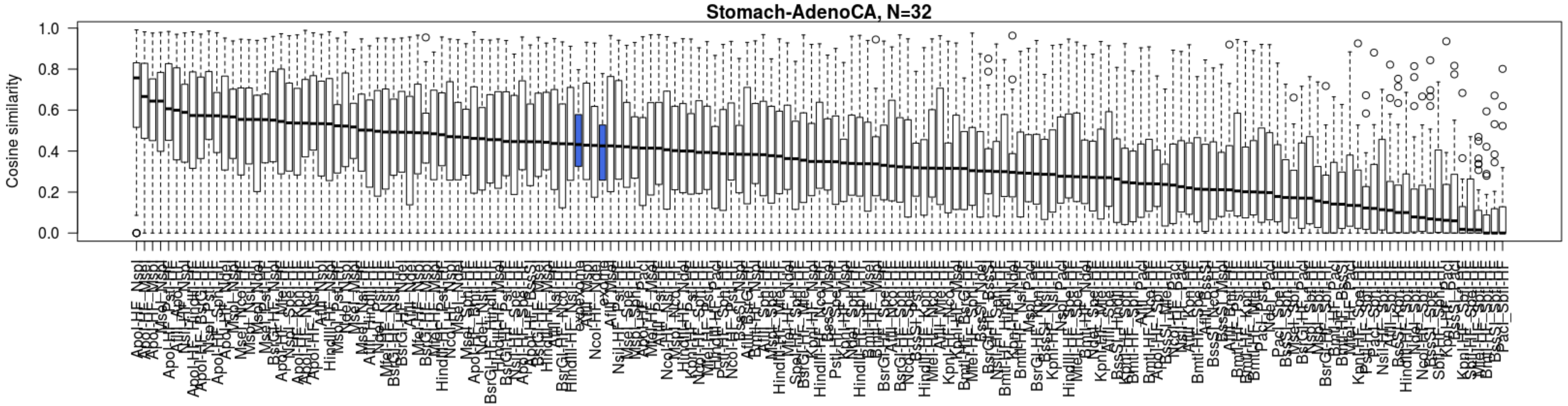
Stomach-AdenoCA, N=32
Cosine similarity
1.0
0.8
0.6
0.4
0.2
0.0

## Supplementary Figure 1: The efficiency of RR-seq-based mutational calling across the PCAWG tumor types.

A) The distribution of cosine similarities between the RR-seq computational simulation-derived (best combination of enzyme per tumor type) and WGS-based mutational signatures. Data are shown as boxplots, where the bold line at the center indicates the median and the upper and lower hinges extend to the 25th and 75th percentile, respectively. The upper/lower whisker extends from the upper/lower hinge to the largest/smallest value no further than 1.5 times the interquartile range from the upper/lower hinge. Samples outside this range are indicated as points. For each cancer type the number of samples per group (N) is indicated within the x-axis labels.

B) Scatterplot of the log10-scaled median number of mutations (x-axis) and the median performance of the RR-seq computational simulation-based mutational signatures measured by cosine similarity to the WGS-based mutational signatures (y-axis) per PCAWG cancer type. Each point represents one cancer type.

C) Mutational signatures computationally simulated across the PCAWG cohort. Summary of the cosine similarities (y-axis) of WGS-derived mutational signatures and mutational signatures derived from subsets of mutations simulating different sequencing approaches (x-axis) for each of the of individual tumor types from the PCAWG cohort. Boxes show the 25% and 75% quartile with the median across the samples indicated by the bold line. Whiskers extend to 1.5 times the interquartile range and samples outside this range are indicated as points. Different enzyme combinations were simulated for RR-seq, each shown as a different box. RR-Seq – reduced representation sequencing, WES – whole exome sequencing, expanded WES – whole exome sequencing expanded to untranslated regions and miRNAs. Title of each page contains abbreviated tumor name (explained in Supplementary Figure 1) and the number of samples used for the analysis.

Abbreviations: Eso-AdenoCa – Esophageal Adenocarcinoma; AdenoCA – Adenocarcinoma; Lymph-BNHL – B-cell Non-Hodgkin Lymphoma; HCC – Hepatocellular Carcinoma; Head-SCC – Head and Neck

Squamous Cell Carcinoma; Panc-AdenoCA – Pancreatic Adenocarcinoma; CNS-Medullo – Medulloblastoma and variants; RCC – Renal Clear Cell adenocarcinoma, papillary type; Myeloid-AML – Acute Myeloid Leukaemia; Bone-Osteosarc – Osteosarcoma; Myeloid-MPN – Myeloproliferative neoplasm; Lymph-CLL – Chronic Lymphocytic Leukaemia; Prost-AdenoCa – Prostate Adenocarcinoma; Bone-Epith – Adamantinoma, Chordoma; Panc-Endocrine – Neuroendocrine carcinoma; CNS-PiloAstro – Pilocytic astrocytoma.

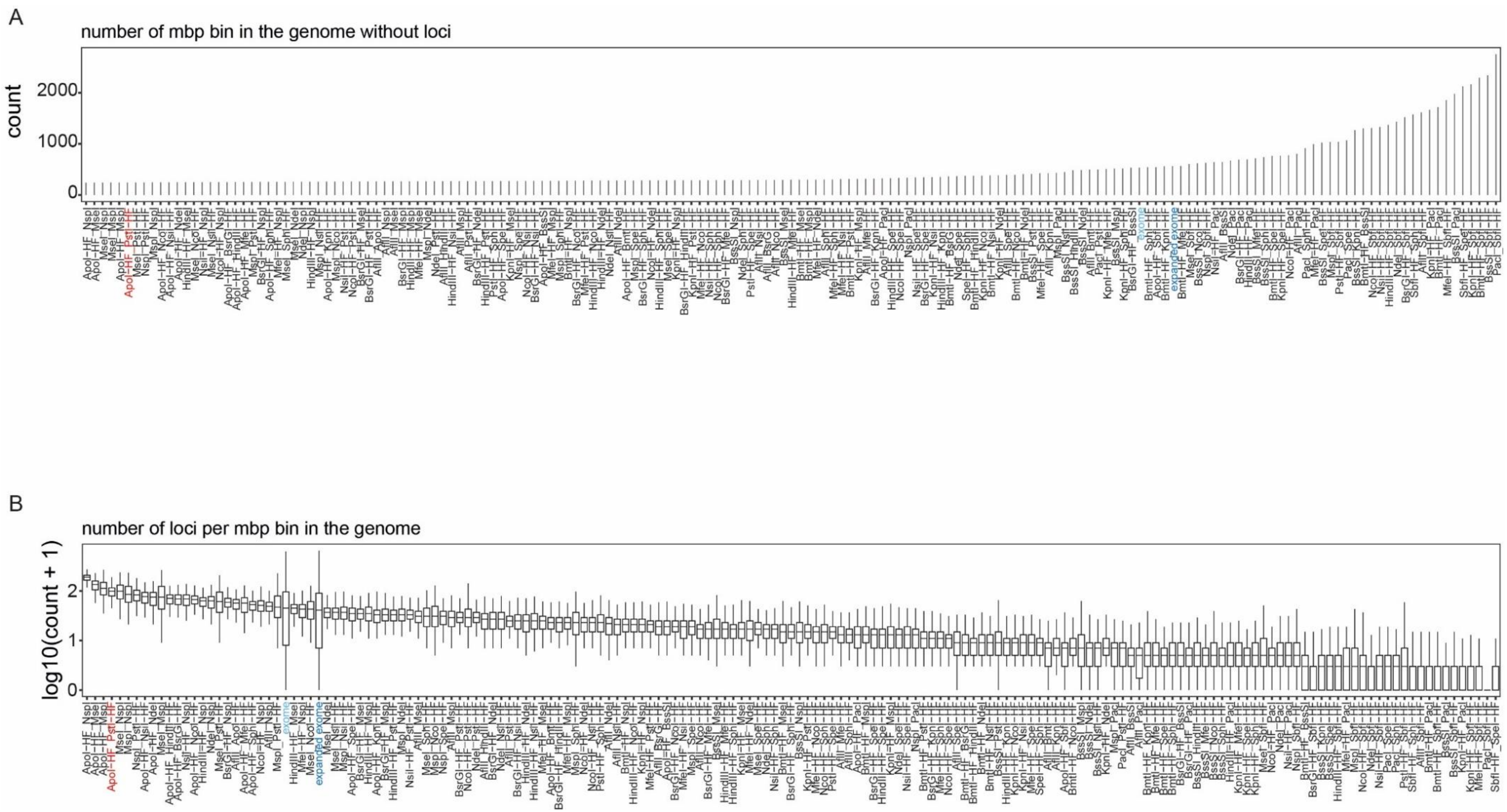
A
number of mbp bin in the genome without loci
count
2000
1000
0
B
number of loci per mbp bin in the genome
log10(count + 1)
2
1
0
exome
expanded exome

## Supplementary Figure 2 – Summaries of the genome-wide distribution of loci resulting from the different sequencing approaches

A) Bar plot of the number of genome-wide consecutive 1Mbps bins that are not covered by at least one expected loci in the computational simulation for each RR-seq with different enzyme combinations and (expanded) WES (x-axis).

B) Summary of the number of expected loci per 1 million base pair (Mbp) bin (n=3211 genome-wide consecutive bins) on logarithmic scale (y-axis) for each RR-seq with different enzyme combinations and (expanded) WES (x-axis). Data are shown as boxplots, where the bold line at the center indicates the median and the upper and lower hinges extend to the 25th and 75th percentile, respectively. The upper/lower whisker extends from the upper/lower hinge to the largest/smallest value no further than 1.5 times the interquartile range from the upper/lower hinge.

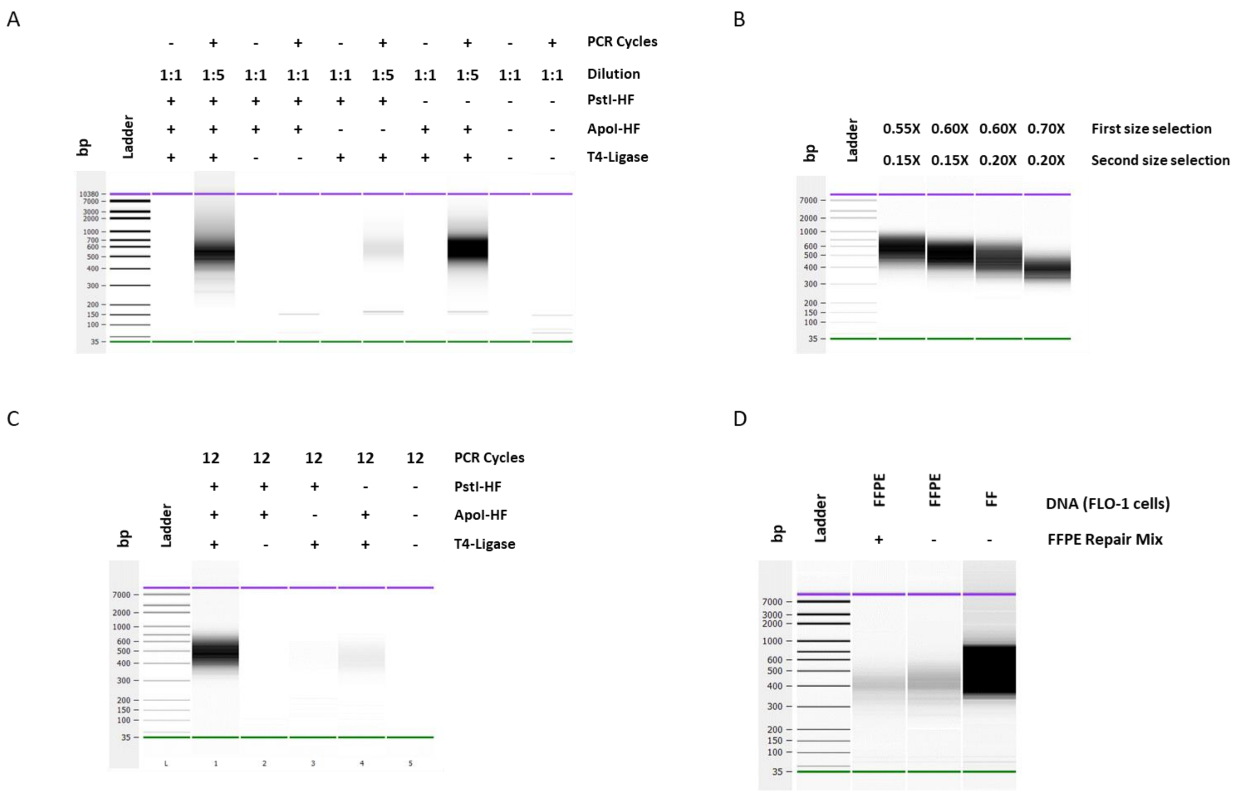


## Supplementary Figure 3 – Optimization of mutREAD library preparation using FLO1 cell line

A) Bioanalyser traces for the optimization of the single step double digestion and ligation. 500 ng of FLO1 genomic DNA was used for ligation of mutREAD adapters in the presence of indicated enzymes and underwent PCR amplification (20 cycles) using Illumina compatible primers. Samples before (-) and after (+) PCR are shown for each enzyme combination. Dilution indicates dilution of samples for bioanalyzer analysis (for samples that exceeded recommended detection range).

B) Bioanalyser traces for different titration of ratios of AMPure beads and ligated DNA solution (50ul) to optimize the double size selection of the fragments in the library.

C) Bioanalyser traces prepared under optimised PCR cycles conditions. Note significant decrease in the level of ApoI only fragments when compared to 20 PCR cycles (A).

D) Bioanalyser traces showing improved bands for FFPE samples after treatment with FFPE repair mix and library preparation with optimized protocol.

All samples were run using DNA High Sensitivity Bioanalyzer kit with standard DNA ladder. Green and purple bands indicate lower and upper markers respectively. The experiments were repeated twice independently to check the reproducibility of optimized conditions.

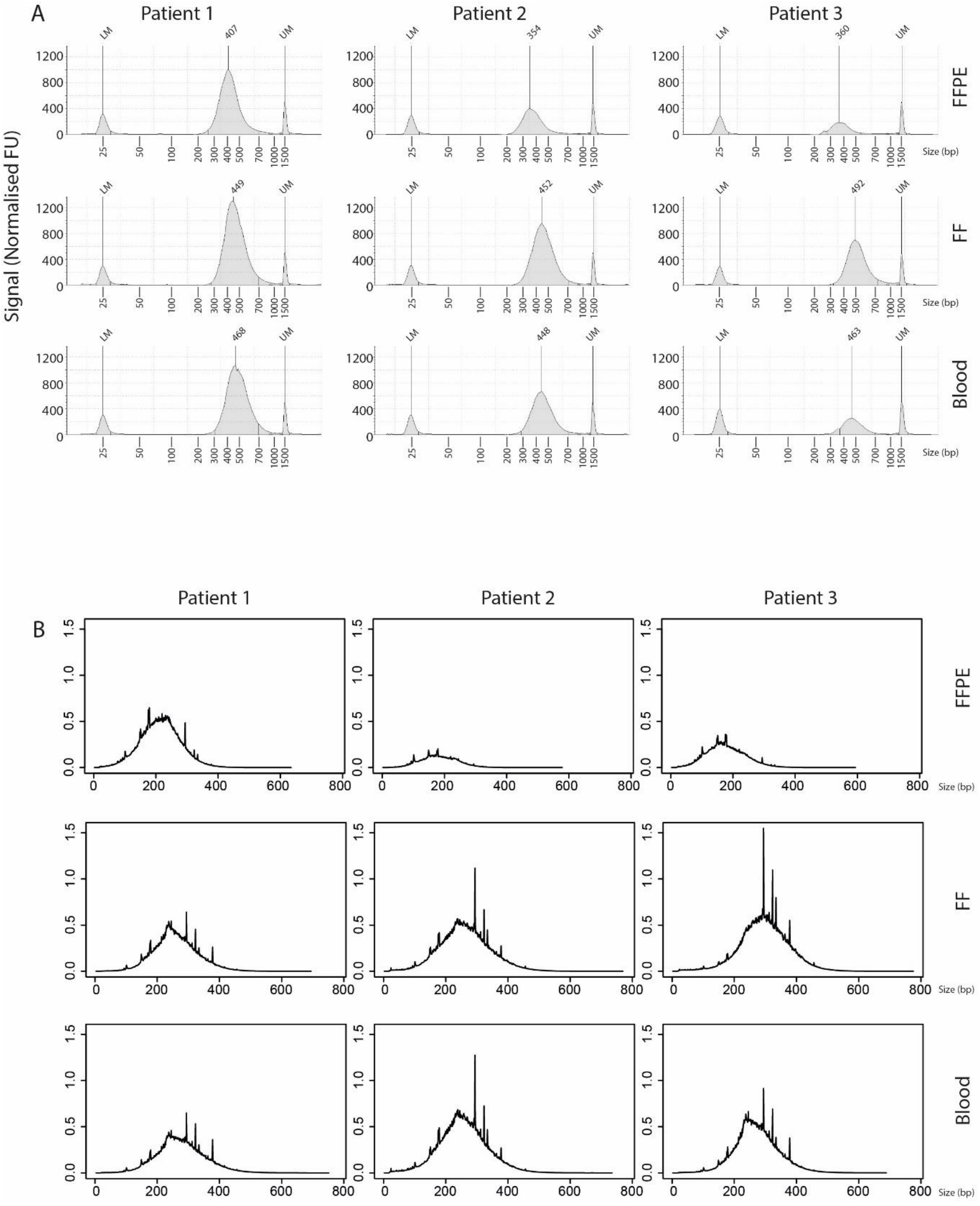


## Supplementary Figure 4 – Comparison of the expected and sequenced fragment size distribution.

A) Fragment size (x-axis) distribution of sequencing libraries measured on the Tape-station. Electropherograms of DNA fragments from three samples derived from FFPE (neat), Fresh Frozen (FF,

1:4 dilution) and matching blood samples (1:4 dilution) with the average size of libraries highlighted above the plot. LM – lower marker, UM – upper marker, FU – fluorescent units.

B) Fragment size distribution derived from read-pairs mapped to the human genome. Each plot shows the number of fragments (y-axis) for each length in base pairs (x-axis). The fragment length was calculated as the number of base pairs between the 5' ends of the read mates (including restriction site parts but not adapters or barcode sequences) and summarized to a histogram using Picard's CollectInsertSizeMetrics function.

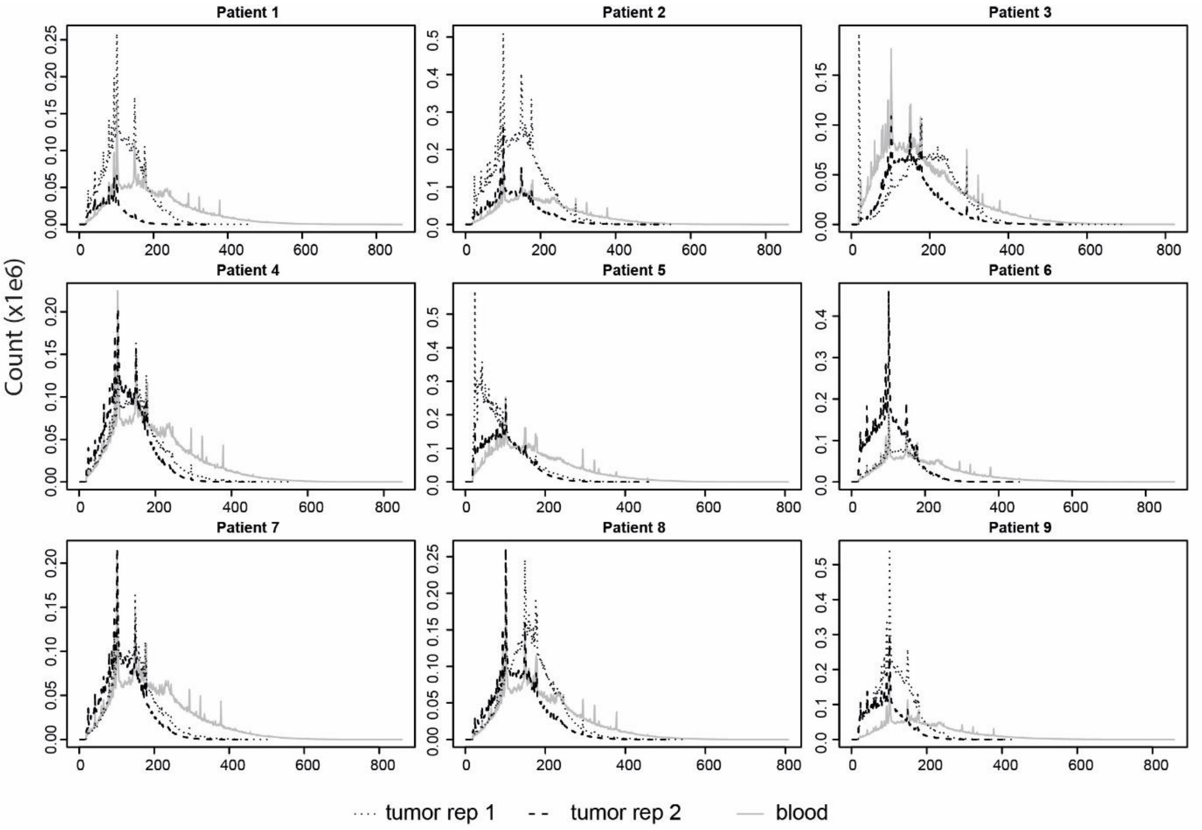


## Supplementary Figure 5 – Comparison of the fragment size distributions for technical replicates of FFPE samples and blood

Fragment size distribution derived from read-pairs mapped to the human genome. Each plot shows the number of fragments (y-axis) for each length in base pairs (x-axis) for the two technical replicates of FFPE tumor samples and the corresponding blood sample per patient. The fragment length was calculated as the number of base pairs between the 5’ ends of the read mates (including restriction site parts but not adapters or barcode sequences) and summarized to a histogram using Picard’s CollectInsertSizeMetrics function.

# Supplementary Tables

**A) mutREAD adapter sequences**

| Adapter | Adapter name | Sequence |
|---|---|---|
| **mutREAD-i5** | mutREAD-i5-upper_1_ATGAGCGA | 5'-C GCT CTT CCG ATC T HNNNATGAGCGATGCA-phos-3' |
| | mutREAD-i5-lower_1_TCGCTCAT | 5'-phos-TCGCTCATNNNDA GAT CGG AAG AGC GTC GTG TAG GGA AAG AGT GT-3' |
| | mutREAD-i5-upper_2_GCCTAGCG | 5'-CGCTCTTCCGATCTHNNNGCCTAGCGTGCA-phos-3' |
| | mutREAD-i5-lower_2_CGCTAGGC | 5'-phos-CGCTAGGCNNNDAGATCGGAAGAGCGTCGTGTAGGGAAAGAGTGT-3' |
| **mutREAD-i7** | mutREAD-i7-upper_1_CGTGTACC | 5'-GTGACTGGAGTTCAGACGTGTGCTCTTCCGATCTHNNNCGTGTACC-3' |
| | mutREAD-i7-lower_1_GGTACACG | 5'-AATTGGTACACGNNNDAGATCGGAAGAGCA-3' |
| | mutREAD-i7-upper_2_GCACATGT | 5'-GTGACTGGAGTTCAGACGTGTGCTCTTCCGATCTHNNNGCACATGT-3' |
| | mutREAD-i7-lower_2_ACATGTGC | 5'-AATTACATGTGCNNNDAGATCGGAAGAGCA-3' |

**B) mutREAD primer sequences**

| Primer | Primer Name | Sequence |
|---|---|---|
| **mutREAD-i5nn** | mutREAD-i501_TATAGCCT | 5'-AATGATACGGCGACCACCGAGATCTACAC**TATAGCCT**ACACTCTTTCCCTACACGAC*G-3' |
| | mutREAD-i502_ATAGAGGC | 5'-AATGATACGGCGACCACCGAGATCTACAC**ATAGAGGC**ACACTCTTTCCCTACACGAC*G-3' |
| **mutREAD-i7nn** | mutREAD-i701_ATTACTCG | 5'-CAAGCAGAAGACGGCATACGAGAT**CGAGTAAT**GTGACTGGAGTTCAGACGTGTGC*T-3' |
| | mutREAD-i702_TCCGGAGA | 5'-CAAGCAGAAGACGGCATACGAGAT**TCTCCGGA**GTGACTGGAGTTCAGACGTGTGC*T-3' |

**Legend**

| | |
|---|---|
| NNNNN | Unique Molecular Identifier |
| NNNNN | Inner sample barcode |
| **NNNNN** | Outer sample barcode |

## Supplementary Table 1 – mutREAD adapters and primers

A) Summary of the sequences of mutREAD adapters used for ligation to DNA fragments. Color of the nucleotides indicates specific elements of the adapters (unique molecular identifiers, inner samples barcodes). Ambiguous base codes H and D translate to bases A/C/T and A/G/T, respectively. Adapter names include the arm of the adapter (i5 contains PstI compatible end and i7 – ApoI compatible end) and the sequence of the samples barcode.

B) Summary of the Illumina compatible primers used for amplification of the ligated libraries. Color indicates the sequences and location of outer sample barcode. Adapter names include the arm of the primer (i5XX is compatible with i5 adapter and i7XX with i7 adapter) and the sequence of the samples barcode.

Abbreviations: phos – phosphorylation of the indicated nucleotide, * - phosphorothioate bond between the indicated bonds.

| Patient ID (1) | Sample type (2) | Number of reads after outer barcode demultiplexing (3) | Percent retained after QC (4) | Percent lost due to unidentifyable barcode (5) | Percent lost due to low quality (6) | Percent of reads lost due to inner barcode mixup (7) | Percent lost due to ambiguous RAD-tag (8) | Estimated average fragment size (bp) (9) |
|---|---|---|---|---|---|---|---|---|
| Tumor1 | **FF** | 158,178,068 | 94.54 | 0.09 | 0.37 | 3.83 | 1.17 | 260 |
| | **FFPE** | 184,526,290 | 93.41 | 0.33 | 0.36 | 4.46 | 1.43 | 215 |
| | **Blood** | 155,543,840 | 94.14 | 0.07 | 0.42 | 3.52 | 1.85 | 276 |
| Tumor2 | **FF** | 206,790,834 | 92.68 | 0.23 | 0.62 | 5.55 | 0.91 | 263 |
| | **FFPE** | 43,949,748 | 94.92 | 0.23 | 0.47 | 3.41 | 0.98 | 183 |
| | **Blood** | 230,847,264 | 96.21 | 0.13 | 0.48 | 2.37 | 0.80 | 257 |
| Tumor3 | **FF** | 231,185,296 | 95.00 | 0.07 | 0.46 | 3.56 | 0.92 | 297 |
| | **FFPE** | 86,259,612 | 94.26 | 0.67 | 0.59 | 3.33 | 1.15 | 178 |
| | **Blood** | 194,066,264 | 96.39 | 0.08 | 0.47 | 2.02 | 1.05 | 273 |
| **Patient ID (1)** | **Sample type (2)** | **Base pairs covered with at least 10x (10)** | **Percent of retained reads contributing to 10x loci (11)** | **Base pairs in 10x loci shared in tumour/blood pair (12)** | **Base pairs covered with at least 50x (13)** | **Base pairs covered with at least 100x (14)** | **mutREAD - Number of mutations (15)** | **WGS - Number of mutations (16)** |
| Tumor1 | **FF** | 175,049,803 | 96.54 | 166,936,824 | 98,935,164 | 60,297,417 | 1,050 | 28,732 |
| | **FFPE** | 170,810,606 | 96.91 | 143,331,473 | 122,274,170 | 86,782,166 | 383 | |
| | **Blood** | 186,266,055 | 96.26 | - | 103,044,362 | 60,323,363 | - | - |
| Tumor2 | **FF** | 195,958,931 | 96.63 | 187,858,494 | 147,765,532 | 105,375,328 | 1,471 | 27,764 |
| | **FFPE** | 95,105,098 | 93.98 | 88,953,041 | 32,115,195 | 10,201,299 | 47 | |
| | **Blood** | 193,634,665 | 96.61 | - | 147,906,131 | 111,689,924 | - | - |
| Tumor3 | **FF** | 198,984,001 | 96.57 | 170,614,310 | 146,079,968 | 106,880,654 | 530 | 11,068 |
| | **FFPE** | 131,586,722 | 95.11 | 113,854,654 | 77,474,870 | 36,663,830 | 90 | |
| | **Blood** | 190,613,393 | 96.49 | - | 114,092,331 | 73,822,016 | - | - |

## Supplementary Table 2 – Quality metrics for mutREAD libraries derived from tumor, FFPE and blood samples of three patients.

The table summarizes quality metrics for each sample, including fresh-frozen (FF) and formalin-fixed paraffin-embedded (FFPE) tumor, as well as blood samples (column 2) from three patients (column 1). Sample groups of three were sequenced on one lane, where each sample had a unique outer barcode. Number of reads derived from the libraries de-multiplexed by outer barcode are listed in column 3. Percentages (with respect to column 3) of reads that are retained for further analysis (column 4) or filtered due to an unidentifiable inner barcode (column 5), low read quality (column 6), wrong/unexpected inner barcode (column 7), missing restriction site overhang (column 8) are listed in the respective columns. The average fragment size derived from read pair mates after mapping is given in column 9 (related to Supplementary Figure 4). The number of base pairs covered with at least 10x, 50x and 100x is listed in column 10, 13 and 14, respectively. The percentage of retained reads (column 4) contributing to loci defined in column 10 is given in column 11. Finally, the overlap between tumor and blood samples in loci defined in column 10 is shown in column 12. The number of mutations used for deriving the mutational signatures is given in column 15 and 16.

**A) Number mutations (fresh-frozen)**

| | mutREAD | | | WGS | | |
|---|---|---|---|---|---|---|
| | Mutect2 | Strelka | Consensus | Mutect2 | Strelka | Overlap |
| Tumor1 | 1050 | 520 | 440 | 28732 | 27370 | 26048 |
| Tumor2 | 1471 | 839 | 714 | 27764 | 26540 | 25284 |
| Tumor3 | 530 | 339 | 217 | 11068 | 10398 | 9950 |

**B) Cosine similarity with WGS (fresh-frozen)**

| | Mutect2 | Strelka | Consensus |
|---|---|---|---|
| Tumor1 | 0.96 | 0.94 | 0.92 |
| Tumor2 | 0.95 | 1.00 | 0.99 |
| Tumor3 | 0.96 | 0.84 | 0.83 |

**C) Number mutations (FFPE)**

| | Mutect2 | Strelka | Consensus |
|---|---|---|---|
| Tumor1 | 383 | 811 | 104 |
| Tumor2 | 47 | 420 | 27 |
| Tumor3 | 90 | 838 | 45 |

**D) Cosine similarity with WGS (FFPE)**

| | Mutect2 | Strelka | Consensus |
|---|---|---|---|
| Tumor1 | 0.89 | 0.83 | 0.76 |
| Tumor2 | 0.93 | 0.81 | 0.89 |
| Tumor3 | 0.96 | 0.81 | 0.88 |

## Supplementary Table 3 – Comparison of Mutect2 and Strelka mutation calling pipelines

The tables A and C summarize the number of mutations detected by Mutect2 and Strelka, as well as the overlap/consensus between the two mutation callers, for the three fresh-frozen (mutREAD and WGS) and FFPE tumor samples, respectively. The cosine similarity of mutREAD-derived and WGS-derived mutational signatures is summarized in tables B and D for the fresh-frozen and FFPE samples, respectively. For each mutation caller and the consensus set, the mutational signatures were calculated from respective mutREAD-derived and WGS-derived mutation set and compared against each other using cosine similarity.

A) 10x sWGS

| Patient ID | Sample type | Number of reads after outer barcode demultiplexing | Properly paired reads | Base pairs covered with at least 10x | Percent of retained reads contributing to 10x loci | Base pairs in 10x loci shared in tumour/blood pair | Number of mutations |
|---|---|---|---|---|---|---|---|
| Tumor1 | **FF** | 215,680,416 | 206,317,606 | 2,432,340,493 | 90.21 | 99,329,518 | 42 |
| | **Blood** | 180,158,855 | 175,855,924 | 2,248,030,642 | 88.49 | - | - |
| Tumor2 | **FF** | 329,742,782 | 321,605,192 | 2,646,459,519 | 88.55 | 77,553,550 | 21 |
| | **Blood** | 217,304,771 | 210,403,566 | 2,444,354,485 | 88.51 | - | - |
| Tumor3 | **FF** | 139,225,587 | 134,967,944 | 1,914,933,524 | 89.19 | 94,152,562 | 83 |
| | **Blood** | 233,793,837 | 228,289,614 | 2,478,664,018 | 88.27 | - | - |

B) WES

| Patient ID | Sample type | Number of reads after outer barcode demultiplexing & PCR clone removal | Properly paired reads | Base pairs covered with at least 10x | Percent of retained reads contributing to 10x loci | Base pairs in 10x loci shared in tumour/blood pair | Number of mutations |
|---|---|---|---|---|---|---|---|
| Tumor1 | **FF** | 149,007,546 | 146,883,752 | 228,030,295 | 92.41 | 396,961,129 | 325 |
| | **Blood** | 72,706,935 | 69,490,692 | 165,989,267 | 89.08 | - | - |
| Tumor2 | **FF** | 44,956,340 | 44,224,012 | 119,724,582 | 94.68 | 902,341,754 | 142 |
| | **Blood** | 64,528,333 | 63,067,844 | 145,330,607 | 92.69 | - | - |
| Tumor3 | **FF** | 74,142,650 | 72,864,976 | 156,263,110 | 82.16 | 251,229,586 | 46 |
| | **Blood** | 84,750,728 | 79,661,310 | 187,169,707 | 92.76 | - | - |

C) mutREAD

| Patient ID | Sample type | Number of reads after outer barcode demultiplexing, PCR clone removal & mutREAD filtering | Properly paired reads | Base pairs covered with at least 10x | Percent of retained reads contributing to 10x loci | Base pairs in 10x loci shared in tumour/blood pair | Number of mutations |
|---|---|---|---|---|---|---|---|
| Tumor1 | **FF** | 150,115,473 | 142,432,706 | 175,049,803 | 96.54 | 166,936,824 | 1,050 |
| | **Blood** | 145,505,593 | 137,261,472 | 186,266,055 | 96.26 | - | - |
| Tumor2 | **FF** | 196,879,399 | 195,040,496 | 195,958,931 | 96.63 | 187,858,494 | 1,471 |
| | **Blood** | 219,749,023 | 217,691,576 | 193,634,665 | 96.61 | - | - |
| Tumor3 | **FF** | 216,676,031 | 214,418,984 | 198,984,001 | 96.57 | 170,614,310 | 530 |
| | **Blood** | 185,303,214 | 175,401,460 | 190,613,393 | 96.49 | - | - |

## Supplementary Table 4 – Quality metrics for 10x sWGS, WES and mutREAD libraries derived from tumor and blood samples of three patients.

The table summarizes quality metrics of the libraries generated by (A) 10x sWGS, (B) WES, and (C) mutREAD for tumor and blood samples (column 2) of the same three patients (column 1). Column 3 and 4 gives the number of reads and properly paired read pairs used for mutation calling, respectively. The number of base pairs covered with at least 10x, the percentage of reads contributing to these loci and the overlap in these loci between tumor and normal samples is listed in column 5-7.

**A) Tumor biopsies**

| Patient | Pathologist 1 | Pathologist 2 | Pathologist 3 |
|---|---|---|---|
| Tumor1 | 70% | 60% | 45% |
| Tumor2 | 50% | 55% | 30% |
| Tumor3 | 30% | 35% | 15-20% |

**B) Tumor resections**

| Patient | Pathologist 1 | Pathologist 2 | Pathologist 3 |
|---|---|---|---|
| Patient1 | 60% | 60% | 15% |
| Patient2 | 60% | 70% | 20% |
| Patient3 | 30% | 60% | 15% |
| Patient4 | N/A | N/A | N/A |
| Patient5 | 30% | 20% | 10-15% |
| Patient6 | 70% | 40% | 20% |
| Patient7 | 20% | 45% | 20-25% |
| Patient8 | 25% | 45% | 50% |
| Patient9 | 50% | 50% | 20-25% |

## Supplementary Table 5 – Tumor cellularity of FFPE samples estimated by pathology

A) Estimated percent of tumor content for the three biopsy samples estimated by pathologist review of diagnostic slides.

B) Estimated percent of tumor content for the nine tumor resection samples estimated by pathologist review of diagnostic slides.

| A) mutREAD | | | | | |
|---|---|---|---|---|---|
| **No.** | **Reagents** | **Size** | **Cost (£)** | **Use per sample** | **Per sample (£)** |
| 1 | Adapter and Primers | 400ul | -- | 9.5ul | 2 |
| 2 | T4 ligase | 20,000Units | 62.58 | 400units | 1.25 |
| 3 | ApoI-HF | 1000 Units | 49.6 | 50units | 2.48 |
| 4 | PstI-HF | 10,000Units | 41.6 | 50units | 0.2 |
| 5 | 10mM ATP | 1000ul | 24 | 4ul | 0.1 |
| 6 | Ampure XP beads | 5000ul | 195.26 | 37.5ul | 1.5 |
| 7 | 10mM dNTPs | 800ul | 49.6 | 2ul | 0.1 |
| 8 | Phusion High fidelity polymerase | 100Units | 61.6 | 1unit | 0.6 |
| | | | | **Total:** | **£8.23** |
| | | | | | |
| **B) WES** | | | | | |
| **Sl no** | **Reagents** | **Size** | **Cost (£)** | **Per sample (£)** | |
| 1 | DNA library preparation and enrichment kit | 16 | 3,589 | 199 | |
| | | | **Total:** | **£199** | |
| | | | | | |
| **C) 10x sWGS** | | | | | |
| **Sl no** | **Reagents** | **Size** | **Cost (£)** | **Per sample (£)** | |
| 1 | Thruplex library preparation and enrichment kit | 96 | 3,818.59 | 39.7 | |
| 2 | Sonication | 96 | 352 | 3.7 | |
| | | | **Total:** | **£43** | |

## Supplementary Table 6 – Comparative cost evaluation for library preparation per sample

A) Estimated cost of individual elements used for library preparation using the mutREAD protocol. The cost is estimated using reagents provided by New England Biolab, Ipswich, MA 01938 USA

B) Cost estimate of enrichment-based whole exome sequencing provided by Agilent, Santa Clara, CA 95051 USA. The cost does not include AMPure XP and Streptavidin beads required for the selection of target sequences.

C) Cost estimate of the whole genome library preparation method provided by Takara Bio, Kusatsu, Shiga 525-0058, Japan. The cost does not include AMPure XP and Streptavidin beads required for the selection of target sequences.

Cost of sequencing: Assuming 200x coverage for mutREAD, the per-sample cost is around £150. The costs for WES would be similar as both methods sample a comparable proportion of the genome. 10x sWGS would cost £300-£700 depending on the chosen sequencing platform.

| Restriction enzyme | Restriction site sequence |
|---|---|
| AflII | C*TTAAG |
| ApoI | R*AATTY |
| BmtI | GCTAG*C |
| BsrGI | T*GTACA |
| BssSI | C*ACGAG |
| HindIII | A*AGCTT |
| KpnI | GGTAC*C |
| MfeI | C*AATTG |
| MseI | T*TAA |
| MspI | C*CGG |
| NcoI | C*CATGG |
| NdeI | CA*TATG |
| NsiI | ATGCA*T |
| NspI | RCATG*Y |
| PacI | TTAAT*TAA |
| PstI | CTGCA*G |
| SbfI | CCTGCA*GG |
| SpeI | A*CTAGT |
| SphI | GCATG*C |

## Supplementary Table 7 – List of restriction enzymes tested in the computational simulation and their restriction site sequences

The table lists the enzymes selected as described in the Methods section and their restriction sites (5'→3'), with the cutting position indicated by *, highlighting the different possible overhangs. Ambiguous codes R and Y translate to A/G or C/T, respectively, and indicate that either base at this position is accepted by the enzyme.

| Patient ID | Anonymized Name (ICGC identifier) | Age at diagnosis | Sex | Tumor Stage | Node Stage |
|---|---|---|---|---|---|
| Tumor1 | a504c27a1fd7af3a53e0b5108cc052cab5ff8d1a353800e85ea8eec766707bde | 75.4 | male | T3 | N0 |
| Tumor2 | fa37be85256c1efbac0501eb13ae1daf9f0fcaff4a7cbb3d2f4420f70f75d334 | 77.1 | male | T3 | N1 |
| Tumor3 | da859c8e95cc5acefde4e70aaed8fc89449c2aa5d9a6cef6202041987840e0a3 | 75.3 | male | T2 | N1 |
| Patient1 | 9a498e8b17034fd8bb534f0e65e12c83a73aa65908fd41f4a13f86cf35b3e0cd | 80.8 | female | T1a | Nx |
| Patient2 | 493bf7322b8c18365466c43bf7a9e119bd4d7782147f9bc368bf4909539c43de | 68.7 | male | T1b | N0 |
| Patient3 | 078773cc36a9ab58ece5c92e50368462dea52ded70f1b0da8c66e65066a3ce53 | 72.4 | male | T3 | N1 |
| Patient4 | 3180f8e34845d13e27dddd90486dba083cd87a340d919f75d29859766f7faee4 | 69.9 | male | T3 | N1 |
| Patient5 | a7376de8be895a08d2abb22b1e3ee2483fb47abc534d7c6a066b06b2f0d4459a | 51.9 | female | T1 | N0 |
| Patient6 | 75ce1bd6dbaf2d4ca50f51e6c1f2a09f3275b080539a07ac7ed962ae72c5179f | 58.4 | female | T3 | N0 |
| Patient7 | 1395dc4ab7c754e0a84c8daa3996f16e5caf11169f8a9be6c800f6da00474321 | 65.6 | male | T2 | N1 |
| Patient8 | 934fe84809fc20a81f124747d5ed57817eb0f9120ea63a69a1548d514710978f | 59.2 | male | T4a | N1 |
| Patient9 | 900fdae05c90e27aba521996cc05d0a83e32dec27c271a8f14e59fa84439ed34 | 73.5 | male | T3 | N0 |

## Supplementary Table 8 – Patients Clinical Characteristics

The table lists the information about individual patients used in the study. Patient ID follows the convention established in figures 1 and 2. Anonymized Name provides ICGC patient ID that can be used to obtain the Whole Genome Sequencing data used in the study.
