# Supplementary material for: The mutREAD method detects mutational signatures from low quantities of cancer DNA
Source: Nat Commun. 2020 Jun 23;11:3166. doi: 10.1038/s41467-020-16974-3 (PMC7311535; doi:10.1038/s41467-020-16974-3)
Supplement: Supplementary file 3 — Reporting Summary [file 41467_2020_16974_MOESM3_ESM.pdf]

nature research

Corresponding author(s): Rebecca C. Fitzgerald

Last updated by author(s): May 21, 2020

Reporting Summary

Nature Research wishes to improve the reproducibility of the work that we publish. This form provides structure for consistency and transparency in reporting. For further information on Nature Research policies, see Authors & Referees and the Editorial Policy Checklist .

Statistics

For all statistical analyses, confirm that the following items are present in the figure legend, table legend, main text, or Methods section.

n/a	Confirmed	
☐	☒	The exact sample size (*n*) for each experimental group/condition, given as a discrete number and unit of measurement
☐	☒	A statement on whether measurements were taken from distinct samples or whether the same sample was measured repeatedly
☒	☐	The statistical test(s) used AND whether they are one- or two-sided<br>*Only common tests should be described solely by name; describe more complex techniques in the Methods section.*
☒	☐	A description of all covariates tested
☒	☐	A description of any assumptions or corrections, such as tests of normality and adjustment for multiple comparisons
☐	☒	A full description of the statistical parameters including central tendency (e.g. means) or other basic estimates (e.g. regression coefficient) AND variation (e.g. standard deviation) or associated estimates of uncertainty (e.g. confidence intervals)
☒	☐	For null hypothesis testing, the test statistic (e.g. *F*, *t*, *r*) with confidence intervals, effect sizes, degrees of freedom and *P* value noted<br>*Give P values as exact values whenever suitable.*
☒	☐	For Bayesian analysis, information on the choice of priors and Markov chain Monte Carlo settings
☒	☐	For hierarchical and complex designs, identification of the appropriate level for tests and full reporting of outcomes
☒	☐	Estimates of effect sizes (e.g. Cohen's *d*, Pearson's *r*), indicating how they were calculated

Our web collection on statistics for biologists contains articles on many of the points above.

Software and code

Policy information about availability of computer code

Data collection	No software used for data collection.
Data analysis	Tapestation software (v3.2); ddRADseqTools (v0.45); Stacks (v1.46); BWA MEM (v0.7.15); samtools (v1.3.1); GATK callableLoci (v3.7-0); Picard (v2.9.0); SomaticSignatures (v2.14); R(v3.4.2); Strelka (v 2.0.15); GATK Mutect2 (v3.7-0), custome code is availabe at https://github.com/jperner/mutREAD

For manuscripts utilizing custom algorithms or software that are central to the research but not yet described in published literature, software must be made available to editors/reviewers. We strongly encourage code deposition in a community repository (e.g. GitHub). See the Nature Research guidelines for submitting code & software for further information.

Data

Policy information about availability of data

All manuscripts must include a data availability statement. This statement should provide the following information, where applicable:

- Accession codes, unique identifiers, or web links for publicly available datasets
- A list of figures that have associated raw data
- A description of any restrictions on data availability

All mutREAD data generated for the article will be available from European Genome-phenome Archive (accession number EGAD00001006170). WGS data for the matched patient samples is available from the ICGC data portal (https://dcc.icgc.org/, information about the patient ID are provided in Supplementary Table 8).

Field-specific reporting

Please select the one below that is the best fit for your research. If you are not sure, read the appropriate sections before making your selection.

Life sciences study design

All studies must disclose on these points even when the disclosure is negative.

Sample size	Sample sizes for each experiment are clearly delineated in the manuscript. No statistical methods were used to predetermine sample size. For the simulated data, we used all available WGS data set. For the demonstration of the efficiency of the mutational signature capture by our method, we used sample size in line with previous studies that demonstrated the use of the similar methods.
Data exclusions	The exclusion criteria were not pre-established and were established during exploratory data analysis and are delineated in the relevant sections of the Methods.
Replication	Findings that are highlighted int eh manuscript were successfully reproduced using independent methodologies (WGS, exome sequencing, mutREAD). For all patient-based mutREAD analysis, each patient was analyzed once. For the optimization experiments, all assays were run as two independent replicates.
Randomization	No randomization was used as there was no treatment in our study.
Blinding	Blinding was not possible, as knowledge of the experimental conditions was required during the data collection and analyses.

Reporting for specific materials, systems and methods

We require information from authors about some types of materials, experimental systems and methods used in many studies. Here, indicate whether each material, system or method listed is relevant to your study. If you are not sure if a list item applies to your research, read the appropriate section before selecting a response.

Materials & experimental systems

n/a	Involved in the study	
☒	☐	Antibodies
☐	☒	Eukaryotic cell lines
☒	☐	Palaeontology
☒	☐	Animals and other organisms
☐	☒	Human research participants
☒	☐	Clinical data

Methods

n/a	Involved in the study	
☒	☐	ChIP-seq
☒	☐	Flow cytometry
☒	☐	MRI-based neuroimaging

Eukaryotic cell lines

Policy information about cell lines

Cell line source(s)	FLO-1 cell line used in this study was derived from a distal oesophageal adenocarcinoma (1991) from a 68-year, male patient with Caucasian ethnicity. It is hypodiploid (1.9) and commercially available from culture collection of Public Health England.
Authentication	The Cell line was authenticated using STR profiling.
Mycoplasma contamination	Cell were tested negative for mycoplasma contamination.
Commonly misidentified lines (See ICLAC register)	No cell lines used are commonly misidentified in the ICLAC register

Human research participants

Policy information about studies involving human research participants

Population characteristics	Esophageal adenocarcinoma samples were collected by the Oesophageal Cancer Classification and Molecular Stratification (OCCAMS) project, a multi-center UK-wide study. The clinical data about the individual patients is included in Supplementary Table 8.
Recruitment	Patients are recruited after diagnosis of oesophago-gastric cancer and samples taken at times of clinically indicated interventions either at the time of surgery or before using biopsies. Our small cohort of patients was randomly selected from a large cohort of adenocarcinoma patients. We cannot rule out patient selection bias, however these biases should not have effect on the results as pair-wise (for individual patients) analysis was performed.
Ethics oversight	Esophageal adenocarcinoma samples were collected by the Oesophageal Cancer Classification and Molecular Stratification (OCCAMS) project, a multi-center UK-wide study. The study was approved by the East of England - Cambridge South Research Ethics Committee (REC 07/H0305/52 and 10/H0305/1) and included individual informed consent.

Note that full information on the approval of the study protocol must also be provided in the manuscript.
